# Genetic diversity of *Anopheles stephensi* in Ethiopia provides insight into patterns of spread

**DOI:** 10.1186/s13071-021-05097-3

**Published:** 2021-12-11

**Authors:** Tamar E. Carter, Solomon Yared, Dejene Getachew, Joseph Spear, Sae Hee Choi, Jeanne N. Samake, Peter Mumba, Dereje Dengela, Gedeon Yohannes, Sheleme Chibsa, Matthew Murphy, Gunawardena Dissanayake, Cecilia Flately, Karen Lopez, Daniel Janies, Sarah Zohdy, Seth R. Irish, Meshesha Balkew

**Affiliations:** 1 Department of Biology, Baylor University, Waco, TX USA; 2 Department of Biology, Jigjiga University, Jigjiga, Ethiopia; 3 Dire Dawa University, Dire Dawa, Ethiopia; 4 USAID, Addis Ababa, Ethiopia; 5 Abt Associates, PMI VectorLink Ethiopia Project, Addis Ababa, Ethiopia; 6 Abt Associates, PMI VectorLink Project, Rockville, MD USA; 7 U.S President’s Malaria Initiative (PMI) Program, Addis Ababa, Ethiopia; 8 USAID, Bureau for Global Health, Office of Infectious Disease, Malaria Division, 2100 Crystal Drive| 10082B, Arlington, VA 22202 USA; 9 Department of Bioinformatics and Genomics, University of North Carolina at Charlotte, Charlotte, NC USA; 10 U.S. President’s Malaria Initiative and Entomology Branch, Centers for Disease Control and Prevention, Atlanta, GA USA

**Keywords:** Malaria, Invasive species, Horn of Africa, Sequencing, Phylogeography, Vector-borne

## Abstract

**Background:**

The recent detection of the South Asian malaria vector *Anopheles stephensi* in the Horn of Africa (HOA) raises concerns about the impact of this mosquito on malaria transmission in the region. Analysis of *An. stephensi* genetic diversity and population structure can provide insight into the history of the mosquito in the HOA to improve predictions of future spread. We investigated the genetic diversity of *An. stephensi* in eastern Ethiopia, where detection suggests a range expansion into this region, in order to understand the history of this invasive population.

**Methods:**

We sequenced the cytochrome oxidase subunit I (*COI*) and cytochrome B gene (*CytB*) in 187 *An. stephensi* collected from 10 sites in Ethiopia in 2018. Population genetic, phylogenetic, and minimum spanning network analyses were conducted for Ethiopian sequences. Molecular identification of blood meal sources was also performed using universal vertebrate *CytB* sequencing.

**Results:**

Six *An. stephensi COI-CytB* haplotypes were observed, with the highest number of haplotypes in the northeastern sites (Semera, Bati, and Gewana towns) relative to the southeastern sites (Kebridehar, Godey, and Degehabur) in eastern Ethiopia. We observed population differentiation, with the highest differentiation between the northeastern sites compared to central sites (Erer Gota, Dire Dawa, and Awash Sebat Kilo) and the southeastern sites. Phylogenetic and network analysis revealed that the HOA *An. stephensi* are more genetically similar to *An. stephensi* from southern Asia than from the Arabian Peninsula. Finally, molecular blood meal analysis revealed evidence of feeding on cows, goats, dogs, and humans, as well as evidence of multiple (mixed) blood meals.

**Conclusion:**

We show that *An. stephensi* is genetically diverse in Ethiopia and with evidence of geographical structure. Variation in the level of diversity supports the hypothesis for a more recent introduction of *An. stephensi* into southeastern Ethiopia relative to the northeastern region. We also find evidence that supports the hypothesis that HOA *An. stephensi* populations originate from South Asia rather than the Arabian Peninsula. The evidence of both zoophagic and anthropophagic feeding support the need for additional investigation into the potential for livestock movement to play a role in vector spread in this region.

**Graphical Abstract:**

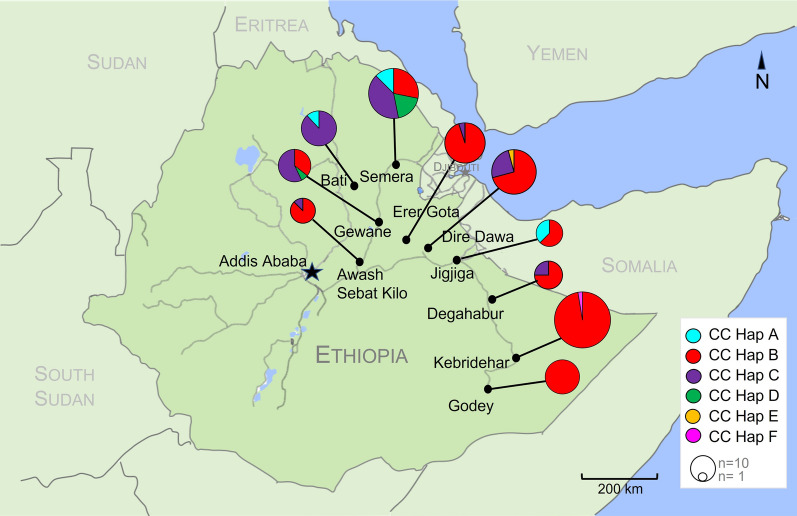

**Supplementary Information:**

The online version contains supplementary material available at 10.1186/s13071-021-05097-3.

## Background

Malaria remains one of the leading global health concerns, with over 229 million cases reported yearly [1]. Efforts to prevent the transmission of malaria often involve controlling the mosquito vectors (*Anopheles*) that transmit the malaria parasite, *Plasmodium* spp. Previous studies have shown that the movement of *Plasmodium* strains from one region to another can have serious population health consequences, such as the emergence of antimalarial resistance [2] or malaria epidemics in naïve or low-transmission areas [3]. Investigations into the long-distance movement of these new *Plasmodium* strains have typically centered on the movement of their human hosts as carriers of the strains, especially asymptomatic individuals who act as reservoirs for *Plasmodium* transmission (for review, see Bousema et al. [4]). With the growing evidence that vectors can be transported long distances [5], and given the correlation between new vector introductions and increased disease burden in some instances [6–8], investigating the movement of vector populations in malaria-endemic regions is critical to understanding the introduction of new parasite strains and evaluating the malaria risk in a particular region.

The Horn of Africa (HOA), comprising Djibouti, Eritrea, Ethiopia, and Somalia, is classified as a malaria-endemic area, with the distinction compared to most other areas in Africa of having substantial transmission of both *Plasmodium falciparum* and *Plasmodium vivax* [1]. In 2012, an urban malaria vector mosquito, *Anopheles stephensi,* which is common in South Asia, was detected in the HOA for the first time in Djibouti [9, 10], and then in 2016 in eastern Ethiopia [11]. Previously, this species range was believed to be limited to South Asia and the Middle East, including the eastern Arabian Peninsula [12]. Like *Anopheles arabiensis*, the major malaria vector in the HOA, *An. stephensi* is known to transmit both major malaria parasite species, *P. falciparum* and *P. vivax*, and could threaten recent progress in reducing the prevalence of malaria in that area. After additional surveillance, *An. stephensi* was also detected in the Republic of Sudan and Somalia (Vector Threat Map, WHO, 2019) and was revealed to have a broader distribution in Ethiopia [13] and Djibouti [10], suggesting that *An. stephensi* may spread to other parts of Africa. With molecular evidence that various *Plasmodium* strains evolve to be compatible with specific *Anopheles* species, it was previously unclear whether *An. stephensi* could transmit local HOA *Plasmodium* strains. There is now evidence that the HOA *An. stephensi* can transmit the local strains of *P. falciparum* and *P. vivax* in Djibouti [10] and Ethiopia [14], validating concern for the role *An. stephensi* may play in increasing malaria transmission.

The expansion of *An. stephensi* in the HOA complicates malaria control in the HOA in several ways. Until now, vector control strategies have centered on the detection and elimination of known major vectors like *An. arabiensis* which exhibit different breeding, feeding, and resting behaviors when compared to *An. stephensi*. Also, there is evidence of diverse insecticide resistance mechanisms among *Anopheles* species [15–17]. Given these differences, vector control approaches are in the process of being modified to account for the presence of *An. stephensi* in the HOA. The potential threat to progress in controlling malaria led the World Health Organization (WHO) to post a “vector alert” in 2019, characterizing *An. stephensi* as a “major potential threat,” and calling for enhanced surveillance in Africa [18].

In order to better assess the potential impact of *An. stephensi* on malaria in the HOA and the rest of the continent, more information is needed on the pattern of spread of *An. stephensi*. Analysis of the genetic variation in *An. stephensi* populations can provide crucial information about the geographical range of the species and the frequency of introductions. Phylogeographic and population genetic analyses of the structure of variation and relationships between newly detected *An. stephensi* and *An. stephensi* from long-established populations outside of Africa can provide insight into the evolution of this population in Africa. Initial analysis of the mitochondrial *COI* (cytochrome c oxidase subunit I, also known as *cox1*) generated from *An. stephensi* in Kebridehar in eastern Ethiopia and *An. stephensi* sequence data from collections in South Asia, the Middle East, and the Arabian Peninsula taken from GenBank [11] identified a single haplotype that was different from Djibouti sequences but identical to one *An. stephensi* sequence from Pakistan. Since the identification of *An. stephensi* in Ethiopia, this vector has been detected in 10 additional sites in eastern Ethiopia as far north as Semera and as far south as Godey [13]. Additional data from these sites are needed to provide a more complete picture of the history of *An. stephensi* in Ethiopia. In addition to phylogeographic history, more information on factors that facilitate *An. stephensi* spread is needed. One potential factor of interest is the host preferences of *An. stephensi* in the HOA. Pastoralism is a common livelihood in Ethiopia and involves moving cattle, goats, sheep, and camels hundreds of kilometers every season [19]. Movement may be driven by several factors including limited natural resources, conflicts, recurrent severe droughts, and extreme weather [20, 21]. Resource depletion in recent years has led to movement of livestock over longer distances in search of grazing areas and water [22, 23]. *Anopheles stephensi* has exhibited both zoophagic (common livestock species such as cattle) and anthropophagic behaviors in populations in South Asia [24]. Data on *An. stephensi* feeding preferences in Ethiopia can provide insight into the potential for extensive livestock movement as a vehicle for *An. stephensi* spread. Here we performed phylogeographic and population genetic analyses of *An. stephensi* at 10 sites and used sequence barcoding approaches to evaluate hosts on which *An. stephensi* feeds, to better understand vector movement.

## Methods

### Sample collection and site description

Sample collection was conducted in 10 sites from September to November of 2018 in eastern and northeastern Ethiopia including Semera, Bati, Awash Sebat Kilo, Gewane, Dire Dawa, Erer Gota, Degehabur, Jigjiga, Kebridehar, and Godey as previously detailed [13]. These sites were selected based on the variation in landscape (altitude and topography) and proximity to major roads. Study sites were assigned to categories (northern, central, and southern) based on proximity to specific major roads and relative location across sites. “Northern” sites included the northernmost sites off the B11 road (which runs between Mille town and Kombolcha town) and A1 road (which runs between Ethiopia’s capital, Addis Ababa, and Djibouti). “Central” sites were located in the middle portion of our collection sites along the A10 road (which runs between Addis Ababa and Degehabur) running west/east. “Southern” sites were the southernmost sites off the A10 road running north/south. Mosquitoes were collected using Centers for Disease Control and Prevention (CDC) light traps and pyrethrum spray collection (PSC) in houses, and larvae and pupae were sampled using the WHO dipping approach. CDC light traps were set up from 18:00 to 6:00 local time. Pyrethrum spray collections were performed between 6:00 and 8:00. Larvae and pupae were collected from suspected larval habitats including human-made containers using a standard dipper (350 ml capacity) and pipettes. Adults were reared from collected larvae and morphologically identified to species using palps, wings, abdomen, and legs based on standard identification keys [25–27] and confirmed molecularly through *COI* and *ITS2* sequencing. Overall, a total of 187 mosquitoes were analyzed in this study: 118 wild-caught reared from immatures and 69 wild-caught adults (Table 1).

**Table 1:** Population genetic statistics from collection sites in Ethiopia based *COI* and *CytB* sequences

Sites of collection in eastern Ethiopia	Number	Number of polymorphic (segregating) sites, *s*	Number of haplotypes, *h*	Haplotype diversity, *H*	Nucleotide diversity, *Pi*	Average number of nucleotide differences, *k*
Northern	63	4	4	0.612	0.00148	1.401
Semera	32	4	4	0.728	0.00174	1.647
Bati	17	2	2	0.221	0.00023	0.221
Gewane	14	4	3	0.582	0.00176	1.659
Central	60	4	4	0.345	0.00093	0.877
Awash Sebat Kilo	8	3	2	0.25	0.00079	0.75
Erer Gota	20	3	2	0.1	0.00032	0.3
Dire Dawa	24	4	3	0.453	0.00133	1.257
Jigjiga	8	2	2	0.536	0.00113	1.071
Southern	64	4	3	0.121	0.00032	0.304
Degehabur	12	3	2	0.409	0.0013	1.227
Kebridehar	38	1	2	0.053	0.00006	0.053
Godey	14	0	1	0	0	0
All	187	5	6	0.51	0.00145	1.37

### Analysis of *An. stephensi* DNA

Molecular analyses of specimens were conducted at Baylor University. Two *An. stephensi* genes were selected for genetic diversity analysis. *COI* was chosen based on the availability of sequences from other countries for global phylogenetic analysis. *CytB* was chosen for its successful application in characterizing other Culicidae. While ITS2 was helpful for species identification [28], no genetic variation was observed at this locus in these Ethiopian samples [3] and thus ITS2 phylogenetic analysis was not undertaken. *COI* sequences were generated as previously described [2]. For *CytB*, two primers were designed with Primer3 software [29, 30]: CytbF 5′AGGATCTTCTACAGGACGAG3′ and CytbR 5′CATGTAGGACGAGGAGTCTA3′ and used for polymerase chain reaction (PCR) amplification. Final reagent concentrations and components were 0.4 μM for each primer, 1× Promega GoTAQ Hot Start Master Mix (Promega, Madison, WI, USA), and water for a total reaction volume of 25 µl. The temperature protocol was performed as follows: 94 °C for 5 min, 35 cycles of 94 °C for 40 s, 56 °C for 1 min, 72 °C for 3 min, and final extension of 72 °C for 10 min. Amplification was confirmed using visualization of a 750 bp band with gel electrophoresis. PCR products were sequenced using Sanger technology with ABI BigDye™ Terminator v3.1 chemistry (Thermo Fisher, Santa Clara, CA) according to the manufacturer’s recommendations and run on an Applied Biosystems 3130 Genetic Analyzer (Thermo Fisher, Santa Clara, CA, USA). *CytB* sequences were trimmed and analyzed using CodonCode Aligner v. 8.0.2 (CodonCode Corporation, Centerville, MA, USA). To confirm amplification of the correct locus, sequences were submitted as queries to the National Center for Biotechnology Information's (NCBI) Basic Local Alignment Search Tool (BLAST) [31] against the nucleotide collection in the NCBI GenBank under default parameters [max high-scoring segment pairs (HSP) 250, expect threshold 10, word size 28, optimized for highly similar hits, not specific to any organism]. Sequences of the haplotypes identified in this study are available on the NCBI Nucleotide Database (Accession# OK663479-OK663484, OK670743-OK670744).

### Population genetic analysis

To estimate the level of diversity of the *An. stephensi* collection overall and within each site, we calculated the number of polymorphic (segregating) sites (*s*), number of haplotypes (*h*), haplotype diversity (*H*), nucleotide diversity (*Pi*), and average number of nucleotide differences (*k*) using the DnaSP version 5 program [32]. Genetic diversity statistics were also generated for each collection site subregion, designated “northern,” “central,” and “southern,” as listed in Table 1. Population differentiation between sites was determined through pairwise *F*_st_ (differentiation based on haplotype frequencies only) and *Φ*_st_ (differentiation accounting for genetic distances) in Arlequin version 3.5.2.2 software [33]. To test the significance of the derived pairwise *F*_st_, 110 permutations were performed and *P*-values generated. Significance was set at alpha < 0.00001.

### Phylogenetic analysis

In order to determine the evolutionary relationships between the *An. stephensi* found in Ethiopia and to further elucidate the spread of the vector, we also performed phylogenetic analysis of the *COI* and concatenated *COI*+*CytB An. stephensi* sequences generated in this and previous studies.

Alignments were created with MAFFT version 7 [34] and uneven ends were trimmed using Mesquite 3.51 [35]. Phylogenetic relationships were inferred using RAxML (Randomized Axelerated Maximum Likelihood) [36], which is based on a maximum likelihood (ML) approach. The GTRGAMMA option that uses the general time-reversible (GTR) model of nucleotide substitution with the gamma model of rate of heterogeneity was applied. One thousand replicates were performed with the strategy of searching for the heuristically-best-scoring tree and bootstrap analysis in one run. Best scoring trees under ML with bootstrap values from RAxML were viewed in FigTree v1.4.4 [37] and labeled based on geographical location.

To provide some preliminary insight into the relationship between the haplotypes and other global haplotypes, we also performed phylogenetic analysis using the *COI* sequence data available in GenBank (Additional file 1: Table S1). Analyses were performed as above except that we only incorporated unique haplotypes into the final RAxML analysis. Trees were visualized in FigTree and symbols were added to represent the countries.

### Minimum spanning networks

To evaluate the frequency and relationship between Ethiopian *An. stephensi* haplotypes observed, we generated minimum spanning networks [38] based on the concatenated *COI*-*CytB* sequences using PopART [39]. Similarly, we generated a network of global populations using *COI* sequences from population data sets available in GenBank. Population data sets were only available for Sri Lanka, Pakistan, and Saudi Arabia; analysis was limited to these sets.

### Molecular blood meal analysis

DNA was extracted from abdomens of 59 wild-caught adult blood-fed *An. stephensi* using Qiagen DNeasy kits and was used for molecular identification of blood meal sources. We used a sequence-based approach based on the *CytB* gene and a universal vertebrate-specific primer set to first (i) confirm whether there was detectable vertebrate DNA present in the sample and then (ii) identify the vertebrate host based on querying the NCBI nucleotide sequence database. This primer set can be used to distinguish a broad range of vertebrate species including human, cow, and goat [40] (Kent and Norris 2005). The primers used were UNFOR403 5′TGAGGACAAATATCATTCTGAGG3′ and UNREV1025 5′GGTTGTCCTCCAATTCATGTTA3′. PCR was performed using the following final reagent concentrations and components: 0.4 μM for each primer, 1× Promega GoTAQ Hot Start Master Mix (Promega, Madison, WI, USA), and water for a total reaction volume of 25 µl. Amplification conditions were as follows: 95 °C for 5 min, 35 cycles of 95 °C for 1 min, 58 °C for 1 min, 72 °C for 1 min, and final extension of 72 °C for 10 min. PCR products were confirmed using gel electrophoresis as detailed above, and sequenced and compared with database sequences using BLAST for species identification.

## Results

### Ethiopian *An. stephensi* are genetically diverse

We were interested in the number of haplotypes that would be detected in *An. stephensi* in our study. Analysis of *COI* sequences from 187 *An. stephensi* revealed four segregating sites (polymorphisms) across the sequence that contributed to six distinct *COI*-haplotypes. All polymorphisms were synonymous mutations. *CytB* had a single segregating site resulting in two haplotypes. Genetic diversity statistics were generated for each site including number of polymorphic (segregating) sites (*s*), number of haplotypes (*h),* haplotype diversity (*H*), nucleotide diversity (*Pi*), and average number of nucleotide differences (*k*) (Table 1). Semera, located in the northern portion of eastern Ethiopia, was the most diverse (*h* = 4, *H* = 0.728) and Godey in the south was the least diverse (1 haplotype, *H* = 0). When comparing regions, northern sites had the greatest level of diversity (*h* = 5, *H* = 0.63) and the southern sites had the least (*h* = 3, *H* = 0.121).

### *Anopheles stephensi* in Ethiopia exhibit some population differentiation

**Fig. 1:**
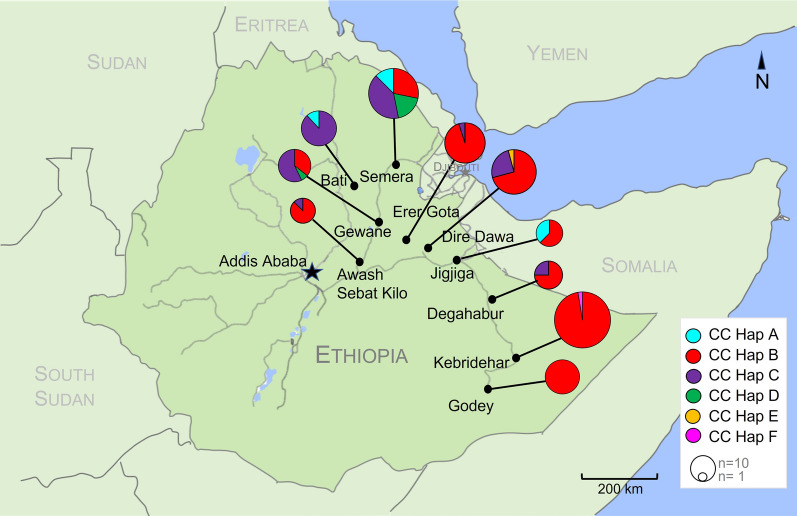
Distribution of *An. stephensi COI-CytB* (CC) haplotypes (Hap) per site in eastern Ethiopia, November–December 2018. Pie charts are proportional to the sample size. Each color represents a different haplotype. Map was created using Adobe Illustrator version 2019 (Adobe, San Jose, CA, USA) and Pages for iOS 13 (Apple Inc., Cupertino, CA, USA) based on maps from Google Maps (https://www.google.com/maps)

**Fig. 2:**
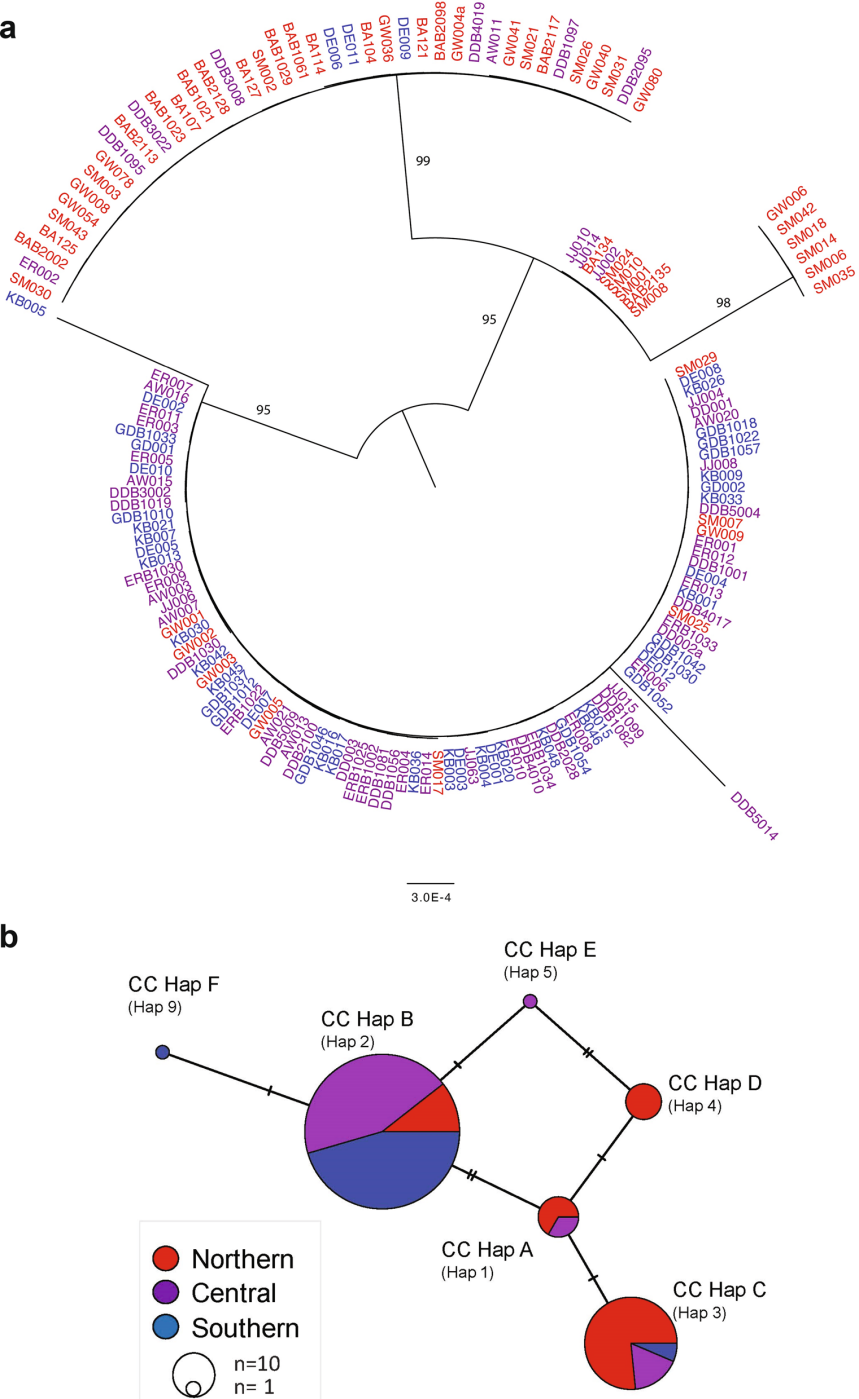
Relationship between *An. stephensi COI-CytB* sequences in Ethiopia. Colors represent subregions with eastern Ethiopia collections. **a** Phylogenetic tree of *An. stephensi COI-CytB* sequences using the maximum likelihood approach based on the general time-reversible substitution model with a gamma model of rate of heterogeneity. Final ML-optimization likelihood = −1256.785865. Only bootstrap values > 70 are shown on branches. **b** Minimum spanning networking of Ethiopian *An. stephensi COI-CytB* (CC) haplotypes. Each node represents a haplotype and the proportion of that haplotype contributed by each Ethiopian region. The size of the nodes is proportional to the sample size. The ticks between nodes represent the number of nucleotide differences. Corresponding *COI* haplotype labels are shown in parentheses (Hap1–5, Hap9)

In addition to different levels of variation in the northern compared with the southern sites, haplotypes were distributed differently across the three designated subregions (Fig. 1). The most prevalent haplotype in the collection was CC-Hap B, and it was observed in all three subregions. CC-Hap B was also the predominant haplotype in the southern and central subregions (93.8% and 82.9%, respectively). However, in the northern subregion, where the highest number of distinct haplotypes was observed, the predominant haplotype was CC-Hap C (55.6%). Pairwise *F*_st_ and *Φ*_st_ reveal population differentiation among study sites (Additional file 2: Figure S1–S2). The highest differentiation (*F*_st_, *Φ*_st_ > 0.80, *P* < 0.00001) was observed between sites in the north and sites in the other two subregions, notably Bati (northern) compared to Kebridehar (southern, *F*_st_ = 0.893, *Φ*_st_ = 0.964), Godey (southern, *F*_st_ = 0.880, *Φ*_st_ = 0.958), and Erer Gota (Central, *F*_st_ = 0.837, *Φ*_st_ = 0.904). Phylogenetic analysis supports population differentiation between the northern and southern sites with bootstraps > 90 for three major clades that carry haplotypes that vary in frequency across these subregions (Fig. 2a). We also constructed a minimum spanning networking based on the *COI-CytB* haplotypes to further evaluate the relationship between the haplotypes (Fig. 2b). The most central nodes (representing specific haplotypes) were CC-Hap A and CC-Hap B. Each haplotype in the network differed by one or two nucleotides.

### Ethiopian *An. stephensi* share haplotypes with several countries

**Fig. 3:**
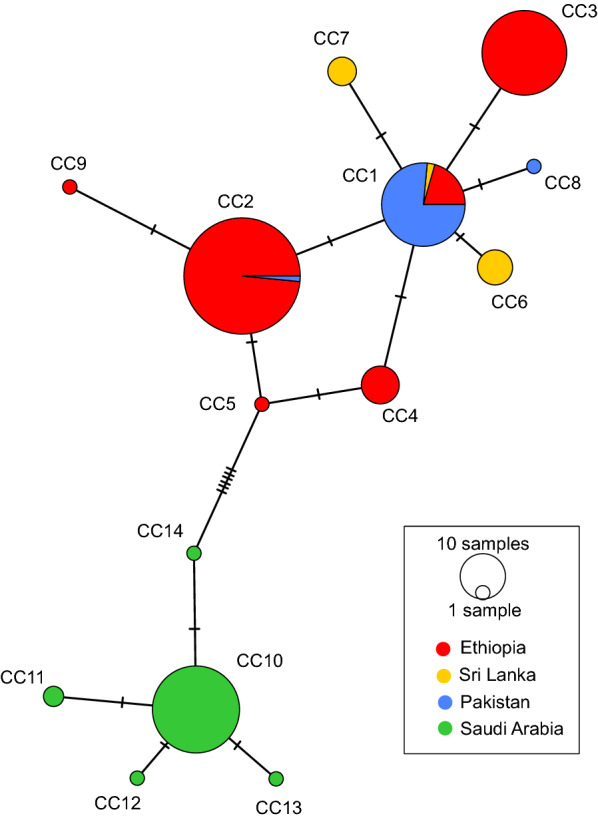
Minimum spanning network of *An. stephensi COI* haplotypes (*H*) from Ethiopia, Sri Lanka, Pakistan, and Saudi Arabia. Each node represents a haplotype and the proportion of that haplotype contributed by each country. The size of the nodes is proportional to the sample size. The ticks between nodes represent the number of nucleotide differences

**Fig. 4:**
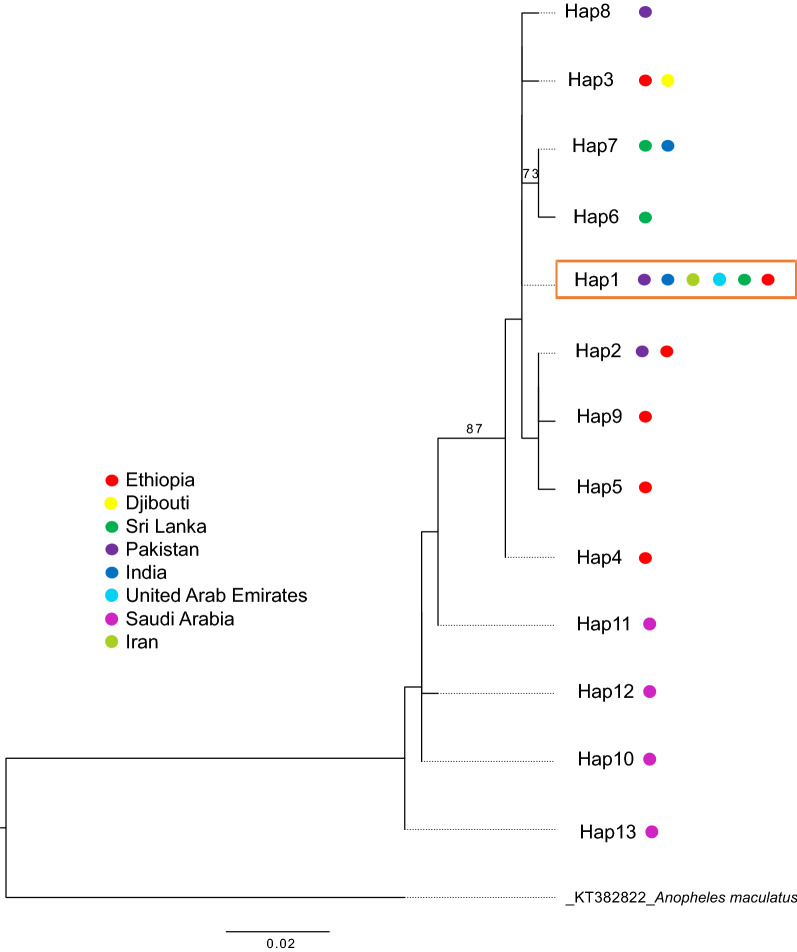
Global *Anopheles stephensi* COI haplotype tree. Each dot represents the country where the COI haplotype had been observed. Only bootstrap values > 70 are shown. The central haplotype with a multi-country distribution is denoted by the orange box

*CytB* sequences were only available for three countries so only *COI* sequences were used for global diversity comparisons. We compared the *COI* sequences from Ethiopian *An. stephensi* sequences to the sequences available in the NCBI GenBank representing countries in South Asia (India, Pakistan, Sri Lanka), the Middle East including Iran and the Arabian Peninsula (Saudi Arabia, United Arabic Emirates), and the HOA (Djibouti, Ethiopia) (see Additional file 1: Table S1 for sequence information). Of the six Ethiopian *COI* haplotypes detected, two were observed in other countries, designated *COI*-Hap1 (all CC-Hap A) and *COI*-Hap2 (all CC-Hap B). The minimum spanning network of the available population *COI* sequence datasets revealed that *COI*-Hap1 was the most central node, with the most proximal nodes representing country-specific haplotypes for Ethiopia, Sri Lanka, and Pakistan that differed from the central node by a single nucleotide (Fig. 3). The haplotype represented by the central node was also the predominant haplotype in Pakistan (26/28). Phylogenetic analysis of the global *COI* haplotypes revealed that the *COI*-Hap1 is the most widely dispersed haplotype, observed in most sites including Djibouti and other locations in the Arabian Peninsula, South Asia, and the Middle East (Fig. 4). Saudi Arabia, with the most basal sequences and strong differentiation from the other *An. stephensi* sequences (bootstrap = 90), lacked the *COI*-Hap1 haplotype and did not share any haplotypes with Ethiopia or any of the other countries (Figs. 3 and 4).

### *Anopheles stephensi* in eastern Ethiopia exhibit zoophilic feeding behaviors

Given the evidence of a recent spread of *An. stephensi* in eastern Ethiopia, we were very interested in whether *An. stephensi* exhibited zoophilic feeding behaviors that may encourage its range expansion through livestock movement. Of the 59 wild-caught adult *An. stephensi* mosquitoes with available DNA from abdomens, 36 (61.0%) were confirmed to be blood-fed based on the presence/absence PCR assay for vertebrate DNA. These originated in Semera (*n* = 27), Erer Gota (*n* = 4), Gewane (*n* = 2), Kebridehar (*n* = 2), and Godey (*n* = 1). Of the 36 blood-fed *An. stephensi*, 75.0% (27/36) had vertebrate DNA identifiable with sequencing. Of the 27 blood meals, 22 were from goat, three from cow, one from human, and one from dog. Of the 22 samples confirmed to have fed on goat, 68.2% (15/22) showed signs of intraspecies mixed feeding based on the presence of double peaks in sequencing data. Of the nine samples considered blood-fed but unidentifiable, four samples showed evidence of cross-species feedings that could not be resolved by sequence analysis, and five samples presented positive presence/absence PCR results for blood-feeding but produced unsuccessful sequencing results. Overall, we found evidence of both livestock and human feeding.

## Discussion

Our analyses revealed multiple mitochondrial gene haplotypes in *An. stephensi* populations in Ethiopia. Higher genetic diversity was observed in the most northern sites, such as Semera, compared to the most southern, such as Godey. Lower genetic diversity can be a signature of a more recent population produced through range expansions or introduction [41–43]. Often, introductions start with a small population with a relatively small amount of genetic diversity, and variation is acquired over time through spontaneous mutation or gene flow. In this study, the observation of higher diversity in the northern sites and lower diversity in the southern sites may indicate that *An. stephensi* in the south represent a more recent population relative to the population in the north. The difference in the level of diversity may also reflect different degrees of gene flow between the *An. stephensi* origin outside of Ethiopia.

We also observed population differentiation between many sites, notably, differentiation of the northern sites from the central and southern sites. These differences may reflect landscape or other natural barriers that minimize gene flow between the populations. Notably, Semera, and Gewane are located in the Rift Valley, which may explain the within-subregion differentiation of Bati from Semera and Gewane. The population differentiation may also reflect geographical differences in *An. stephensi* introduction(s) into Ethiopia. One subregion may have stronger international connectedness which would allow for influx of additional diversity, while another region with lower international connectedness would have lower diversity overall. Further sequencing representative of the genomic variation and the addition of more sites can help identify the relevant demographic and geographical factors that shape the diversity of *An. stephensi*.

Phylogenetic and network analysis (Figs. 3, 4) revealed haplotypes unique to Ethiopia and others observed in other countries. Based on the sequences available, the *An. stephensi* in both Ethiopia and Djibouti are more closely related to *An. stephensi* from South Asia than those from the Arabian Peninsula (with the exception of one sequence from the United Arab Emirates). These results endorse greater support for South Asia as the place of origin for *An. stephensi* in the HOA. However, conclusions about the specific origin (i.e., country) require additional sampling and sequencing from *An. stephensi* populations from countries not well represented in the available database, including Afghanistan, Yemen, Oman, and China, as well as other underrepresented countries in East Africa including Sudan, Djibouti, Eritrea, and Somalia.

The distribution of mitochondrial DNA (mtDNA) haplotypes in Ethiopia may provide insight into how *An. stephensi* was introduced in Ethiopia. The presence of the central *COI*-Hap1 (common in South Asia) at specific sites in Ethiopia may suggest strong connectedness to the source of *An. stephensi* into the HOA. In our analysis, we see the central *COI*-Hap1 haplotype in Semera and Bati in the northern subregion and Jigjiga in the central subregion (Fig. 1). Based on these data, it is possible that *An. stephensi* was introduced into the north and/or central subregions of eastern Ethiopia and subsequently spread into the south. The proximity of these sites to Djibouti and Somalia highlights the importance of additional molecular surveillance in these countries to understand the impact that movement between these countries may have on the spread of *An. stephensi* in the HOA. The presence of multiple haplotypes and the structure of the haplotypes may reflect multiple introductions into the country. What remains unclear is whether the haplotypes unique to Ethiopia in our study emerged in the earliest populations in the HOA and then subsequently spread, or if they were introduced into Ethiopia from populations not represented in this study. Additional genetic analysis from more regions can help answer this question.

These preliminary results provide a framework for continued investigation into the origin of *An. stephensi* in the HOA. Follow-up genetic studies should include the sites in this study with the strongest connection to S. Asia based on the mtDNA analyses completed here as potential sources for introduction into Ethiopia. More genetic analysis is needed that accounts for genome-wide variation and should also focus on underrepresented sites in the HOA and long-established *An. stephensi* populations outside the HOA. Increasing the number of sites will provide a basis for a formal association analysis between geographical location and the level of genetic diversity to test the hypothesis developed here that the *An. stephensi* population in the north is older than the population in the south. Also, additional samples from collections throughout the year could provide important information on seasonal differences in *An. stephensi* diversity.

Combining genetic analysis with relevant data related to anthropogenic and environmental factors would provide a holistic picture of how *An. stephensi* was introduced and could facilitate predicting how the vector could spread further. Studies should look into economic activities related to import and export practices via vehicle, aircraft, and maritime trade. In addition, pastoral activities may be relevant, given the high evidence of feeding on goats, which are common livestock in eastern Ethiopia. More investigations are needed to determine whether the high zoophilic pattern observed in our study is a trait of the HOA *An. stephensi* or is more likely shaped by the availability of livestock compared to humans as a feeding source. Still, the evidence for both zoophilic and anthropophilic feeding in this study supports the need for investigations into whether livestock movement plays a role in the spread of *An. stephensi*. While the livestock themselves are not likely to transport the mosquitoes, they may be an attractant, and the traveling water containers may serve as suitable and mobile breeding habitat. In addition, incorporating climate variables (e.g., rainfall, humidity, temperature, predominant wind pattern and speed) and landscape characteristics would provide additional information on environmental factors that contribute to gene flow.

## Conclusion

Our data on Ethiopian *An. stephensi* genetic diversity suggest a South Asia origin of the HOA *An. stephensi* and a pattern of spread indicating that the southern population in Ethiopia has resulted from a more recent expansion than the northern and central populations. Future studies with expanded genomic analysis will further inform our understanding of migration rates and the role of local adaption on the spread of *An. stephensi* into and throughout the HOA. Collaboration across countries where this mosquito is well established and countries where it is emerging can facilitate a complete understanding of the spread of *An. stephensi* into Africa.

## Supplementary Information

**Additional file 1: Table S1.** List of *COI* sequences retrieved from NCBI included in population genetic and phylogenetic analyses of *An. stephensi*.

**Additional file 2: Figure S1.** Pairwise *F*_st_ between study sites based on genetic distance. *F*_st_ values are plotted below the diagonal and *P*-values above. Color gradient based on *F*_st_ values (yellow = highest, blue = lowest). **Figure S2. **Pairwise *F*_st_ between study sites based on haplotype frequencies. *F*_st_ values are plotted below the diagonal and *P*-values above. Color gradient based on *F*_st_ values (yellow = highest, blue = lowest).

## Data Availability

Data used in this study are included as supplemental files and/or in National Center for Biotechnology Information Nucleotide Database under Accession No. OK663479-OK663484 and OK670743-OK670744.
