# Supplementary material for: Genetic diversity of Anopheles stephensi in Ethiopia provides insight into patterns of spread
Source: Parasit Vectors. 2021 Dec 11;14:602. doi: 10.1186/s13071-021-05097-3 (PMC8665610; doi:10.1186/s13071-021-05097-3)
Supplement: Supplementary file 2 — Additional file 2: Figure S1. Pairwise Fst between study sites based on genetic distance. Fst values are plotted below the diagonal and P-values above. Color gradient based on Fst values (yellow = highest, blue = lowest). Figure S2. Pairwise Fst between study sites based on haplotype frequencies. Fst values are plotted below the diagonal and P-values above. Color gradient based on Fst values (yellow = highest, blue = lowest). [file 13071_2021_5097_MOESM2_ESM.pdf]

Additional file 2: Figure S1. Pairwise F_{st} between study sites based on genetic distance. F_{st} values are plotted below the diagonal and p-values on the top. Color gradient based on F_{st} values (yellow = highest, blue = lowest).

		North			Central				South		
		**Semera**	**Bati**	**Gewane**	**Awash**	**Erer-Gota**	**Dire Dawa**	**Jigjiga**	**Degehabur**	**Kebridehar**	**Godey**
North	**Semera**	.	0.00901	0.53153	0.00901	<0.00001	<0.00001	0.01802	0.02703	<0.00001	<0.00001
	**Bati**	0.24046	.	<0.00001	<0.00001	<0.00001	<0.00001	<0.00001	<0.00001	<0.00001	<0.00001
	**Gewane**	-0.02007	0.25771	.	0.03604	<0.00001	0.02703	0.05405	0.0991	<0.00001	<0.00001
Central	**Awash Sebat Kilo**	0.32193	0.84429	0.31231	.	0.99099	0.59459	0.59459	0.56757	0.1982	0.36937
	**Erer-Gota**	0.47091	0.90401	0.52134	-0.04979	.	0.10811	0.08108	0.28829	0.2973	0.99099
	**Dire Dawa**	0.22546	0.6409	0.19215	-0.04237	0.09245	.	0.45946	0.99099	<0.00001	0.06306
	**Jigjiga**	0.17021	0.75966	0.1861	0.02857	0.25011	-0.00469	.	0.37838	<0.00001	0.04505
South	**Degehabur**	0.21095	0.70119	0.17221	-0.06289	0.10781	-0.06509	-0.02439	.	0.01802	0.0991
	**Kebridehar**	0.6085	0.9641	0.70716	0.17705	0.02137	0.25107	0.55306	0.34638	.	0.99099
	**Godey**	0.50285	0.95792	0.5698	0.07438	-0.01896	0.15185	0.39459	0.20379	-0.03209	.

Additional file 2: Figure S2. Pairwise F_{st} between study sites based on haplotype frequencies. F_{st} values are plotted on the below the diagonal and p-values on top. Color gradient based on F_{st} values (yellow = highest, blue = lowest).

		North			Central				South		
		**Semera**	**Bati**	**Gewane**	**Awash**	**Erer-Gota**	**Dire Dawa**	**Jigjiga**	**Degehabur**	**Kebridehar**	**Godey**
North	**Semera**	.	<0.00001	0.4955	0.01802	<0.00001	<0.00001	0.00901	0.02703	<0.00001	<0.00001
	**Bati**	0.20765	.	0.01802	<0.00001	<0.00001	<0.00001	<0.00001	<0.00001	<0.00001	<0.00001
	**Gewane**	-0.00601	0.2016	.	0.04505	<0.00001	0.07207	<0.00001	0.06306	<0.00001	<0.00001
Central	**Awash Sebat Kilo**	0.23011	0.74062	0.29016	.	0.99099	0.62162	0.18018	0.62162	0.34234	0.46847
	**Erer-Gota**	0.37423	0.83693	0.50024	-0.04979	.	0.04505	0.00901	0.35135	0.99099	0.99099
	**Dire Dawa**	0.14956	0.55201	0.15045	-0.02847	0.11104	.	0.06306	0.99099	<0.00001	0.02703
	**Jigjiga**	0.1645	0.65032	0.27657	0.133	0.32653	0.12352	.	0.15315	<0.00001	0.03604
South	**Degehabur**	0.14644	0.61093	0.15528	-0.06289	0.10781	-0.06218	0.11853	.	0.01802	0.05405
	**Kebridehar**	0.48365	0.89341	0.64568	0.07354	-0.01096	0.22744	0.4844	0.26829	.	0.99099
	**Godey**	0.39861	0.8795	0.54701	0.07438	-0.01896	0.16767	0.39459	0.20379	-0.03209	.
